# VOLUNTEERING AND CHANGES IN CARDIOVASCULAR BIOMARKERS: LONGITUDINAL EVIDENCE FROM THE HEALTH AND RETIREMENT STUDY

**DOI:** 10.1093/geroni/igad104.1611

**Published:** 2023-12-21

**Authors:** Seoyoun Kim, Cal Halvorsen, Sae Hwang Han

**Affiliations:** Texas State University, San Marcos, Texas, United States; Boston College, Boston, Massachusetts, United States; University of Texas at Austin, Austin, Texas, United States

## Abstract

A growing body of research shows that volunteering is beneficial for those served, the volunteers, and the larger communities. However, major challenges remain that hinder the practical implications for volunteer activity as a public health intervention, including potential selection effects, lack of longitudinal studies that adjust for baseline characteristics, and a paucity of studies that consider multiple physical health outcomes in a single model. Using data from the 2006-2016 waves of the Health and Retirement Study (N=18,847) and an outcome-wide approach, the current paper evaluated if changes in volunteering between 2006/2008 (t0) and 2010/2012 (t1) were associated with seven cardiovascular disease biomarkers four years later (2014/2016, t2). Further, these models were adjusted for demographic factors, socioeconomic status, health behaviors, chronic conditions, baseline biomarkers, and baseline volunteering. Inverse probability treatment weights (IPTW) were included for the selection into volunteering and multiple imputation was conducted for missing data. Compared to non-volunteers, volunteering more than 200 hours a year was associated with a lower risk for clinically high diastolic blood pressure. Increased volunteering effort (change from 1-99 hours at t0 to >100 hours at t1) was associated with a lower likelihood of clinically high systolic and diastolic blood pressure levels. Sustained high volunteering (>100 hours at both t0 and t1) was associated with lower diastolic blood pressure. The current study adds to the evidence of the health benefits of volunteering for adults 50 and older by inferring a potential causal link between high-intensity volunteering and reduced blood pressure.

